# Hydrophobic bile acids relax rat detrusor contraction via inhibiting the opening of the Na^+^/Ca^2+^ exchanger

**DOI:** 10.1038/srep21358

**Published:** 2016-02-19

**Authors:** Jingzhen Zhu, Xingyou Dong, Qian Liu, Chao Wu, Qingqing Wang, Zhou Long, Longkun Li

**Affiliations:** 1 Department of Urology, Second Affiliated Hospital, Third Military Medical University, Chongqing 400037, China

## Abstract

Hydrophobic bile acids (BAs) are thought to inhibit smooth muscle contractility in several organs. The present study was undertaken to investigate the effects of hydrophobic BAs on the detrusor contractility of rat bladder and to explore the possible mechanism. Lithocholic acid (LCA) treatment increased the micturition interval and induced a concentration-dependent relaxation of bladder detrusor strips. In addition, LCA reduced the concentration of intracellular free Ca^2+^([Ca^2+^]_i_) and inhibited both the outward and inward Na^+^/Ca^2+^ exchanger (NCX) current (I_NCX_) in primary isolated smooth muscle cells (SMCs). To further investigate the mechanism of action of LCA, several pharmacologic agents were used. We found that the NCX inhibitor 3′,4′-Dichlorobenzamil (DCB) can significantly inhibit the relaxation of detrusor strips and a reduction of the [Ca^2+^]_i_ induced by LCA, while the antagonist of muscarinic receptor and the agonist of the G protein-coupled bile acid receptor (TGR5) and the farnesoid X receptor (FXR) had no effect. In conclusion, these data suggest that the relaxation of rat detrusor induced by hydrophobic BAs is mediated by NCX. Further research is needed to carry out to demonstrate the possible pathway and provide a potential new strategy to investigation for the treatment of the low urinary tract syndromes.

Bile acids (BAs), main component of bile, are synthesized from cholesterol in liver microsomes, where they are conjugated with glycine or taurine^1^. BAs are stored in the gallbladder and secreted into the duodenum and serum in response to feeding. Approximately 95% of BAs are reabsorbed in the intestinal tract by both passive and active transport, and returned back to the liver via the enterohepatic circulation^2^. The enterohepatic circulation is essential for the maintenance of an effective concentration of BAs for cholesterol micellar homeostasis. The mean serum BAs concentration is below 10 μM/L in adult humans, while exceeding 200 μM/L in some pathological conditions^3^. Chenodeoxycholic acid (CDCA) and cholic acid (CA), the two primary BAs in humans, are converted into deoxycholic acid (DCA) and lithocholic acid (LCA), respectively, by bacterial enzymes in the colon. Before BAs are transported out of the hepatocytes, most of them are conjugated to either glycine or taurine. BAs are amphipathic molecules and can be divided into hydrophobic BAs and hydrophilic BAs. The hydrophobic-hydrophilic balance of BAs is determined by biological, aqueous solubility and detergent properties. Previous studies have shown that hydrophobic BAs are the primary BAs in humans, and an excess of hydrophobic BAs is cytotoxic^4^.

BAs, in addition to their classic function in lipid digestion, act as signaling molecules with systemic endocrine functions, such as the regulation of bile acid, glucose and lipid metabolism; the immune response; and cell proliferation and differentiation^2^,^5^. BAs also have direct and/or indirect receptor-mediated effects on multiple processes^6^. BAs regulate glucose and lipid homeostasis by activating both the nuclear receptor farnesoid X receptor (FXR) and the plasma membrane-bound G protein-coupled bile acid receptor 5 (TGR5, also known as GPBAR1)^7^. Modulation of plasma BAs levels and the total BAs pool can affect glycemic control, body weight, and insulin sensitivity^8^.

Moreover, several kinds of BAs are involved in the regulation of the motility of organs. DCA can activate TGR5 to relax the gallbladder smooth muscle and intestinal smooth muscle^9^. Natural bile acids and lithocholate can increase large conductance Ca^2+^-activated K^+^(BK) channel activity, resulting in a relaxation effect on resistance-size arterial smooth muscle and mesenteric artery smooth muscle^10^,^11^. In neonatal rat, a type of BAs, taurocholate (TC) could bind to the muscarinic M_2_ receptor to inhibit the contraction of cultured cardiomyocytes^12^. In the rat, deoxycholyltaurine can reduce the pressure of mesenteric resistance arteries, independent of muscarinic receptor, NO or K^+^ channel activation, and induce the vasodilation of mouse aorta by a M_3_-dependent mechanism^13^. Sodium taurocholate (NaTC) relaxes guinea pig ileal smooth muscle through stimulating the NCX^14^. DCA acts on the L-type calcium current in colonic smooth muscle cells, contributing to a negative intropic effect^15^, and causes vasorelaxation through increasing the extent of lipid peroxidation in vascular tissue^16^.

Considering the important role of BAs in the smooth muscle of gallbladder and intestine, it is necessary to investigate the role of BAs on the bladder, which is a hollow organ composed of smooth muscle and to determine the distribution of BAs. In this study, we investigated the effect of hydrophobic BAs on the detrusor contractility of rat bladder.

## Results

### Effect of LCA on rat bladder voiding

**Figure 1:**
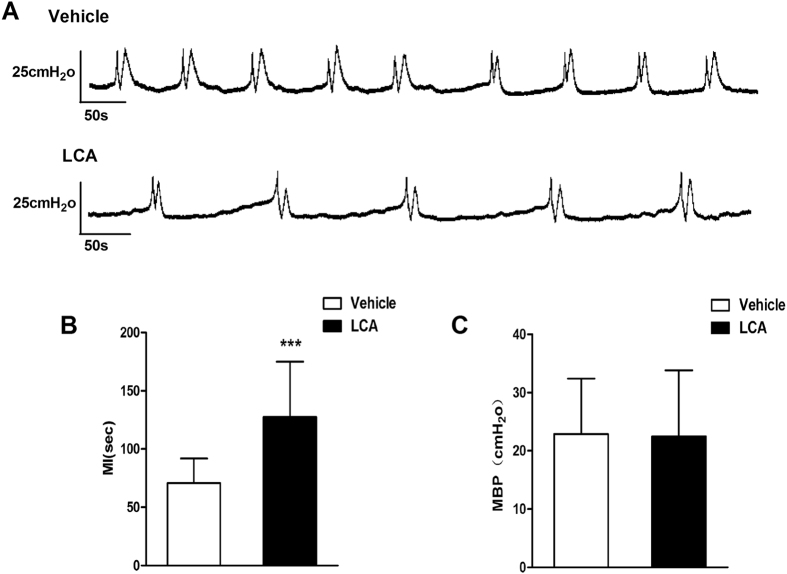
LCA-induced changes in bladder voiding. The voiding interval but not the maximum bladder pressure was changed when LCA (10^−6^ M) was infused into the rat bladder lumen (**A**, n = 5). Compared with the saline vehicle, LCA significantly increased the MI (**B**,127.59 ± 47.40 vs. 70.85 ± 20.95, independent-sample t-test, p = 0.000) but had no influence on MBP (**C**, 22.47 ± 11.34 vs. 22.88 ± 9.51, independent-sample t-test, p = 0.904). ***P < 0.001.

Cystometry was performed to evaluate the effect of LCA on urodynamics in the rat. A continuous pressure curve was recorded when the bladders were perfused with saline and LCA (10^−6^ M) (Fig. 1A). Compared to the saline vehicle, LCA significantly increased the micturition interval (MI; independent-sample t-test, P = 0.000, Fig. 1B). However, LCA did not change the maximum bladder pressure (MBP; independent-sample t-test, P = 0.904, Fig. 1C).

### Relaxation effect of LCA on rat detrusor strips

**Figure 2:**
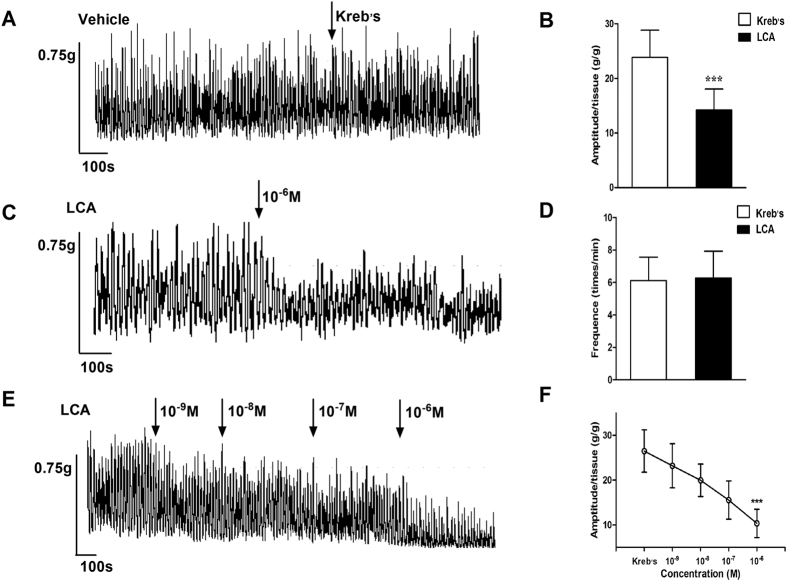
The effects of LCA on the contraction of the rat detrusor strips. Tension recordings revealed the effects of LCA on the contractile activities of the rat detrusor strips (**A**,**C**). Compared with the negative control (n = 6), LCA (10^−6^ M) can decrease the amplitude (**B**, 23.85 ± 5.00 vs. 14.22 ± 3.83, independent-sample t-test, p = 0.000, n = 6), but has no significant effect on the frequency (**D**, 6.29 ± 1.36 vs. 6.34 ± 1.50, independent-sample t-test, p = 0.837, n = 6) of detrusor strip contraction. The dose-response effects of LCA (10^−9^ to 10^−6^ M) were also measured (**E**, n = 5). Gradual decreases in amplitude were observed (**D**, 26.49 ± 4.74 vs. 23.21 ± 4.93 vs. 19.95 ± 3.62 vs. 15.54 ± 4.26 vs. 10.33 ± 3.15, one-way ANOVA, P = 0.000). ***P < 0.001.

The effects of LCA on rat detrusor contraction were detected using detrusor strip tension recording. When LCA (10^−6^ M) was added to the organ bath, the contraction amplitudes were significantly decreased (independent-sample t-test, P = 0.000; Fig. 2B), while the Kreb’s vehicle had no influence on the contraction amplitude (independent-sample t-test, P = 0.771; Fig. 2A). LCA had no effect on the contraction frequency (independent-sample t-test, P = 0.837; Fig. 2D). The concentration-response effects of LCA on detrusor contraction were also detected (Fig. 2E). As LCA concentration increased (from 10^−9^ M to 10^−6^ M), the contraction amplitudes showed a gradient decrease (one-way ANOVA, P = 0.000; Fig. 2F).

### Expression and function of TGR5 in rat bladder

**Figure 3:**
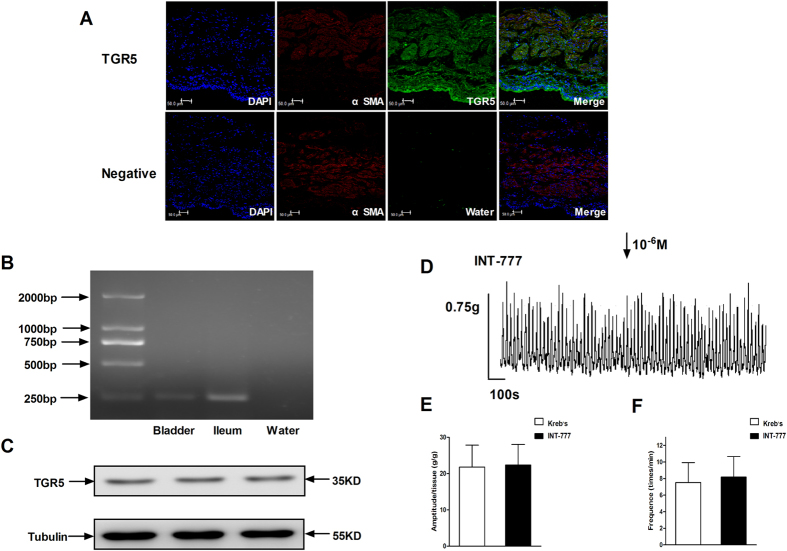
The expression of TGR5 in the rat bladder and the effects of INT-777 on the contraction of the rat detrusor strips. Cross-sections of full-thickness rat bladders show that TGR5 was expressed in the mucosa and muscularis layers. Detrusor cells that express α SMA also express TGR5, as shown by double immunostaining of rat bladders (**A**). The RT-PCR results demonstrated that the TGR5 transcript was present in the rat bladder. Ileum and water were used as positive and negative controls, respectively (**B**). The predicted RNA (or DNA) size was 266 bp. The protein expression of TGR5 was probed with anti-TGR5 using western blotting and anti-tubulin as an endogenous control. The molecular weight of the TGR5 was 35 kDa (**C**). The effects of INT-777 on the contractile activities of the rat detrusor strips were measured (**D**, n = 5). INT-777 did not significantly change the amplitude (**E**, 21.80 ± 6.03 vs. 22.36 ± 5.67, independent-sample t-test, p = 0.639) and the frequency (**F**, 7.55 ± 2.38 vs. 8.20 ± 2.47, independent-sample t-test, p = 0.183) of spontaneous contraction.

The expression of TGR5 mRNA (266 bp) was detected in rat bladders using reverse transcription-polymerase chain reaction (RT-PCR; Fig. 3B). The expression of TGR5 protein (36 kDa) was manifested in rat bladders using western blot analysis (Fig. 3C). In addition, immunofluorescence staining was used to analyze the distribution of TGR5 in rat bladder. The results showed that TGR5 existed in the full-thickness of rat bladder (Fig. 3A). The specific TGR5 receptor agonist INT-777 (10^−6^ M) was used to study the action of TGR5 on detrusor contraction (Fig. 3D). INT-777 did not induce any change on the contraction amplitude (independent-sample t-test, P = 0.639; Fig. 3E) or frequency (independent-sample t-test, P = 0.183; Fig. 3F).

### Roles of the Na^+^/Ca^2+^ exchanger, muscarinic and purinergic receptor, farnesoid X receptor in LCA-induced relaxation on the detrusor strips

**Figure 4:**
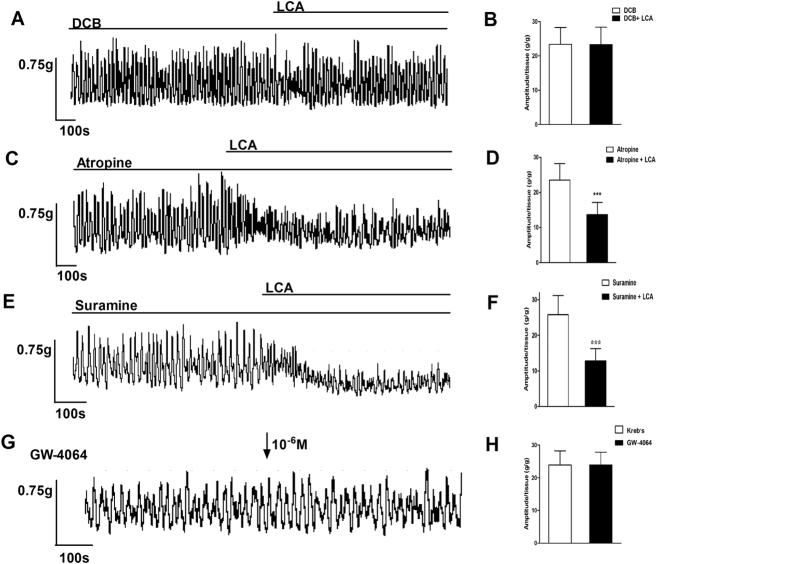
The relationship between LCA and NCX, cholinergic receptor, purinergic receptor, FXR in tension recording. After pretreatment with DCB (10^−5^ M), LCA did not further decrease the contractile amplitude (**A**,**B**, 23.39 ± 4.88 vs. 23.31 ± 5.09, independent-sample t-test, p = 0.933, n = 6). However, after pretreatment with atropine (10^−6^ M), LCA still inhibited the amplitude of spontaneous contraction (**C**,**D**, 23.61 ± 4.53 vs. 13.67 ± 3.46, independent-sample t-test, p = 0.000, n = 6). The same results were found when pretreatment with suramine (10^−6^ M), LCA still inhibited the amplitude of spontaneous contraction (**E**,**F**, 25.79 ± 5.42 vs. 15.38 ± 4.13, independent-sample t-test, p = 0.000, n = 6). GW4064 (FXR agonist, 10^−6^ M) did not significantly change the amplitude of spontaneous contraction (**G**,**H**, 23.91 ± 4.29 vs. 24.12 ± 4.05, independent-sample t-test, p = 0.778, n = 6). ***P < 0.001.

To investigate the possible mechanism of the LCA-induced decrease of contraction amplitude, several pharmacological agents were used in the tension recording to detect the role of the Na^+^/Ca^2+^ exchanger, muscarinic receptor and farnesoid X receptor (Fig. 4A,C,E). After the detrusor strips were pretreated with a blocker of the Na^+^/Ca^2+^ exchanger (DCB, 10^−5^ M) for 10 min, the contraction amplitude was not significantly different when LCA was applied (independent-sample t-test, P = 0.933; Fig. 4B). However, in the detrusor strips pretreated with muscarinic receptor antagonist (atropine, 10^−6^ M) and purinergic receptor antagonist (suramine, 10^−6^ M) for 10 min, the contraction amplitude remained inhibited when LCA was added (independent-sample t-test, P = 000; Fig. 4D). To explore the effect of FXR in LCA-induced relaxation on the detrusor strips, an agonist of FXR (GW-4064, 10^−6^ M) was used (Fig. 4E). The results showed that GW-4064 had no influence on the contraction amplitude of detrusor strips (independent-sample t-test, P = 0.778; Fig. 4F).

### Change in [Ca^2+^]_i_ induced by LCA in primary isolated SMCs

**Figure 5:**
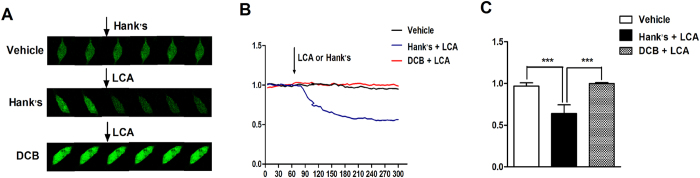
The effects of LCA on intracellular calcium concentration in rat SMCs. Six pictures were chosen to represent the process of instantaneous [Ca^2+^]_i_ changes in the SMCs (**A**, n = 6). The real-time relative fluorescence intensities (RFI, F1/F0) are shown as continuous plots (**B**). Treatment with LCA decreased the [Ca^2+^]_i_ (**C**, 0.99 ± 0.01 vs. 0.64 ± 0.71, independent-sample t-test, P = 0.000), which was drastically abolished by pretreatment with DCB (**C**, 0.64 ± 0.10 vs. 0.97 ± 0.39, independent-sample t-test, P = 0.000). ***P < 0.001.

Fluo-4 AM was used to stain the intracellular calcium ions. The relative fluorescent intensity (RFI) represents the [Ca^2+^]_i_. Continuous images were acquired at a rate of six frames per minute, and six images were chosen as representative of the changing [Ca^2+^]_i_ (Fig. 5A). Compared with the vehicle group, LCA significantly decreased the [Ca^2+^]_i_ (independent-sample t-test, P = 0.000; Fig. 5B,C). When cells were pretreated with DCB, the decrease in [Ca^2+^]_i_ induced by LCA was significantly blocked compared to the Hank’s+LCA group (independent-sample t-test, P = 0.000; Fig. 5B,C).

### Change in NCX current (I_NCX_) induced by LCA in primary isolated SMCs

**Figure 6:**
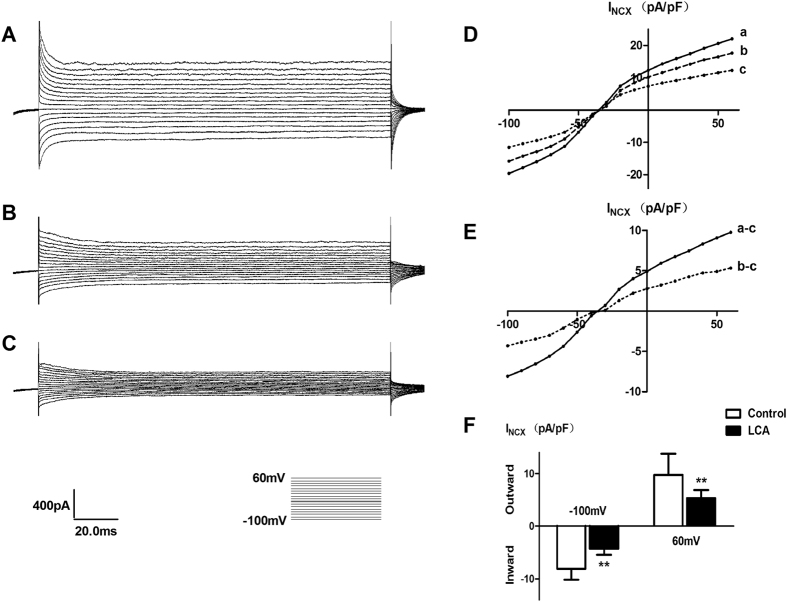
The effect of LCA on I_NCX_ in rat SMCs. (**A**–**C**) represent the typical current curves of the control group, the LCA treated group and the Ni^2+^ treated group. (**D**) represents the I-V curves of three groups (n = 7). I-V curves of net Ni^2+^-sensitive currents were obtained by subtracting the corresponding I-V curves in panel (**D**,**E**). Outward I_NCX_ was significantly inhibited at +60 mV (10.01 ± 3.44 vs. 5.33 ± 1.56, independent-sample t-test, P = 0.006), and inward I_NCX_ was also inhibited at −100 mV (**F**, −8.08 ± 2.08 vs. −4.31 ± 1.11, independent-sample t-test, P = 0.001). **P < 0.01.

I_NCX_ was recorded when SMCs were perfused in extracellular solution (Fig. 6A) and solutions containing 10^−6^ M LCA (Fig. 6B) and 5 × 10^−6^ Ni^2+^ (Fig. 6C), respectively. The I-V curves were constructed (Fig. 6D). The net Ni^2+^-sensitive currents are shown in Fig. 6E. LCA inhibited outward I_NCX_ by 42.82% ± 17.58% at +60 mV (independent-sample t-test, P = 0.006), and inhibited inward I_NCX_ by 46.07% ± 7.84% at −100 mV (independent-sample t-test, P = 0.001, Fig. 6F).

## Discussion

BAs can be divided into hydrophobic BAs and hydrophilic BAs, and have a relaxant effect on the smooth muscle in various organs^15^,^17^,^18^. The relaxant activity of BAs is related to its hydrophobicity; hydrophobic BAs have a greater potential to relax smooth muscle than hydrophilic BAs^19^. In this study, the effects of LCA, a representative hydrophobic BAs, on bladder detrusor contraction were confirmed. We found that LCA can increase the micturition interval and decrease the contraction amplitude of the bladder detrusor and the intracellular calcium concentration. We also investigated the possible mechanism of the LCA relaxant effects. The results showed that the relaxant effects of LCA are dependent on the activation of NCX, but not TGR5, FXR and muscarinic receptors.

For the first time, we demonstrated that LCA (10^−6^ M) could significantly inhibit bladder-voiding contraction. Bladder micturition frequency was decreased after instillation of LCA (10^−6^ M) into the bladder. The effects of LCA on micturition may be related to other mechanisms of the suburothelium. Strong evidence suggests that urothelial cells are involved in bladder sensory functions, which is the important part of the micturition reflex^20^. The changes in bladder excitation induced by LCA require further investigation.

We investigated the effects of LCA on bladder detrusor contractility. The contraction amplitude of rat detrusor strips was significantly decreased. In addition, a concentration-response effect of LCA was also detected in the contraction experiment using detrusor strips. As the LCA concentration increased (10^−9^ to 10^−6^ M), the contraction amplitude gradually decreased, supporting a possible role of LCA on the regulation of bladder contractility. However, the detailed mechanism of action of LCA remains unclear.

Some studies have shown that BAs can decrease the intracellular free Ca^2+^ concentration ([Ca^2+^]_i_)^4^,^15^,^21^ and increase the intracellular Na^+^ concentration^12^. In our experiments, we also found that the [Ca^2+^]_i_ was decreased when the primary isolated SMCs were treated with LCA. Calcium ions are a message transport substance, and play a key role in cell excitation and excitation-contraction coupling^22^. NCX is a nine transmembrane protein and is thought to be a key molecule in the regulation of the homeostatic balance of sodium and calcium. NCX is widely distributed in many types of tissues and involved in various physiological processes^23^. NCX primarily works through two modes: the forward mode (Ca^2+^ exit mode) and the reverse mode (Ca^2+^ entry mode)^24^,^25^. The forward mode of exchange is a normal mode that operates in many physiological conditions. NCX not only can regulate the motility of smooth muscle in many organs and tissues, including cardiac^26^, venous^27^, arterial^28^, tracheal^29^ and ileal^14^, but also has been implicated in various pathological processes^30^. Yamamura *et al.* reported that NCX plays an important role in the pathologic processes of overactive bladder syndromes (OAB)^31^. Fernando *et al.* reported that sodium taurocholate caused a relaxation of isolated guinea pig ileum smooth muscle strips via stimulating the NCX^14^. We next addressed the role of NCX in the relaxation induced by LCA on detrusor strips.

The changes in I_NCX_ induced by LCA were recorded using the whole-cell patch clamp technique. LCA inhibited the outward I_NCX_ by 42.82% ± 17.58% at +60 mV and the inward I_NCX_ by 46.07% ± 7.84% at −100 mV. DCB is a wide-range NCX inhibitor that blocks the antiport in both the forward mode and the reverse mode. When the detrusor strips were pretreated with DCB, LCA no longer decreased the contraction amplitude of detrusor strips. In addition, when the SMCs were pretreated with DCB, the decreased concentration of [Ca^2+^]_i_ induced by LCA was significantly inhibited. These results showed that the NCX is responsible for the inhibitory effects of BAs on detrusor contraction and intracellular calcium mobilization.

TGR5 is a G protein coupled receptor that has been studied in relation to BAs. It was discovered in 2002 and characterized in 2003. TGR5 is activated by multiple BAs, with LCA being the most potent natural agonist. Brigitte *et al.* reported that hydrophobic BAs can act on TGR5 to activate the cAMP-PKA pathway, then opening the K_ATP_ channels to hyperpolarize the membrane and decrease gallbladder smooth muscle activity^19^. Senthilkumar *et al.* reported that activation of TGR5 caused the relaxation of gastric smooth muscle. That relaxation is mediated through the inhibition of the RhoA/Rho kinase pathway via both cAMP/ePac-dependent stimulation of Rap 1 and cAMP/PKA-dependent phosphorylation of RhoA at Ser188^32^. In our study, TGR5 mRNA was detected using RT-PCR, and TGR5 protein was detected using western-blotting and immunofluorescence. We observed that the TGR5 protein was distributed in the full-thickness of rat bladder using immunofluorescence.

To explore the effects of TGR5 on the contraction of detrusor strips, INT-777 (a selective agonist of TGR5) was used. The contraction amplitude and frequency of the detrusor strips were not changed when INT-777 was added to the bath. These results suggest that the activation of TGR5 had no influence on the contraction amplitude and frequency of the detrusor strips. Our results are opposite to the results in other organs^4^,^9^,^19^. This may suggest an organ-specific mechanism, but the detailed mechanism remains to be studied.

We used GW-4064, a selective agonist of FXR, to verify the role of BAs on the nuclear receptor. FXR is a classic nuclear receptor that is activated by BAs^33^. The expression of FXR is more abundant in liver and gut compared to other tissues and is more selectively activated by chenodeoxycholic acid compared to other bile acids. FXR can function as a sensor of metabolic signals and plays an important role in the regulation of bile acid, glucose, and cholesterol homeostasis by controlling the expression of genes related to their metabolism^34^. Morelli, A. *et al.* has reported that FXR is abundantly expressed in the bladder and is involved in the development of metabolic syndrome-induced OAB^35^. In our experiments, the contraction amplitude and the frequency of detrusor strips was not changed when GW4064 was added to the bath. The results indicated that FXR is also not involved in the relaxant effect of hydrophobic BAs. More studies are needed to clarify the role of FXR in OAB.

Cholinergic and purinergic receptors are important signaling pathways regulating smooth muscle contraction, and some studies have reported that BAs might directly bind or indirectly affect muscarinic and purinergic receptor signaling^12^,^36^,^37^. However, other studies found that BAs did not influence muscarinic receptor signaling^38^. In this study, we tried to investigate the interaction of BAs and cholinergic and purinergic signaling in the bladder using detrusor strip tension recording. The detrusor strips were pretreated with a muscarinic and purinergic receptor antagonist (atropine and suramine) before adding LCA. With or without the presence of atropine and suramine, LCA still decreased the contraction of detrusor strips. The results suggest that the relaxation induced by LCA on detrusor strips may be not related to the activation of the muscarinic and purinergic receptors.

In the present study, our results indicated that BAs is involved in the regulation of detrusor contraction, and the NCX may be the key component of downstream molecular pathway. Administration with LCA by intravesical instillation and incubation can influence the bladder micturition and contraction. Therefore intravesical instillation or injection with a purified and synthesized BAs could be developed to modulate overactive detrusor in OAB, the antagonist may be useful for increasing the bladder contractility in diabetes-induced bladder dysfunction.

In conclusion, one limitation of this study is that we did not elucidate possible signal pathways of hydrophobic BAs in low urinary tract syndromes (LUTS). However, this study provides the first evidence of the relaxant effects of hydrophobic BAs on rat bladder detrusor contraction. TGR5, FXR, purinergic and muscarinic receptor have no role in the relaxant effect of hydrophobic BAs, but the activation of NCX may be responsible for the relaxant effect of hydrophobic BAs. Further studies are needed to investigate the expression and functional changes of hydrophobic BAs in LUTS.

## Methods

### Animals

All animal handling and experimental protocols were carried out in accordance with the Guide for the Care and Use of Laboratory Animals, and approved by the Research Council and Animal Care and Use Committee of the Third Military Medical University, China (approval no. SYXK20070002). All efforts were made to minimize animal suffering and to reduce the number of animals used. Fifty-eight adult female Sprague-Dawley rats (190–250 g) were used in our experiments. These rats which were used to measure detrusor strips contraction were killed by cervical dislocation, and the other rats were euthanized by sodium pentobarbital injection (200 mg/kg).

### Detection of TGR5 in the rat bladder by reverse-transcription PCR

Total RNA from rat bladder was extracted in TRIzol reagent, isolated with chloroform, precipitated with isopropyl alcohol, and dissolved in RNAse-free water. For each reaction, 2 μg of total RNA was used to synthesize cDNA using the PrimeScript RT reagent kit (TaKaRa Bio, Tokyo, Japan) and 2 × Taq MasterMix (CWbio, Beijing, China). Ileum tissue and water were used as positive and negative controls, respectively. The primers for rat TGR5 were forward 5′-GAGGGGTTCAGGAGCTTTCC-3′ and reverse 5′-CAGATTGGCAAGCAGGGAGA-3′, generating a fragment of 266 bp.

### Determination of TGR5 in the rat bladder by western blotting

The proteins were extracted for western blotting as we reported previously. Proteins (50 μg) were separated by SDS-PAGE and transferred to nitrocellulose membranes (Immobilon-P^SQ^, Millipore, Billerica, MA, USA). The membranes were blocked with 5% bovine serum albumin (BSA), incubated with primary antibodies, rabbit anti-TGR5 (1:200; ab72608, Abcam, Cambridge, MA, USA) and mouse anti-tubulin (1:500; AT819, Beyotime, Shanghai, China), and incubated with secondary antibody conjugated to horseradish peroxidase (1:2000, Zhongshan Inc., Beijing, China). Immunolabeled proteins were detected by the ChemiDoc XRS + Image System (Bio-Rad Laboratories, Bay Street, CA).

### Identification of the distribution of TGR5 by immunofluorescence staining

The bladders of four rats were fixed in 4% paraformaldehyde (pH 7.4) for 1 h at 4 °C. The tissue sheets were incubated in 1% bovine serum albumin for 2 h at room temperature and then incubated with rabbit anti-TGR5 antibody (1:200) and mouse anti-α-actin antibody (1:400; sc-32251, Santa, California, USA) for 12 h at 4 °C. The samples were washed with phosphate-buffered saline (PBS) and incubated with the secondary fluorescent antibodies conjugated with TRITC and FITC (1:500) for 1 h at room temperature. The samples were washed with PBS and incubated with 4,6-diamidino-2-phenylindole (Sigma-Aldrich, St. Louis, MO) for 10 min at room temperature for nuclear staining. Finally, the samples were imaged using a laser confocal microscope (Leica, Solms, Germany). A procedure identical to that described above was applied to the negative control group with the exception that the rabbit anti-TGR5 antibody was replaced with PBS.

### Measurement of detrusor strip contraction *in vitro*

As previously described^39^,^40^, the rats were euthanized by cervical dislocation. The bladder was carefully removed from each animal and placed in ice-cold Kreb’s solution composed of 119.0 mM NaCl, 4.7 mM KCl, 2.5 mM CaCl_2_, 1.2 mM MgSO4, 25 mM NaHCO3, 1.2 mM KH_2_PO_4_ and 11.0 mM glucose (pH 7.4). The bladder body was longitudinally cut into 3 × 2 × 6-mm strips and placed into a 15-ml organ bath filled with Kreb’s solution. The Kreb’s solution was bubbled with 5% CO_2_ and 95% O_2_ at 37 °C. The strip was then suspended between 2 hooks; one hook was connected to a movable stretch transducer, and the other was fixed to the bottom of the bath. After equilibrating for 30 min, the strips were stretched gradually until the stretch load was maintained at 0.75 g. The continuous curve was recorded with isometric force transducers (Chengyi Co., Chengdu, China). Gradient concentrations of LCA (10^−9^–10^−6^ M) and INT-777 (TGR5 agonist, 10^−6^ M), or GW-4064 (FXR agonist, 10^−6^ M) were added to the bath. In addition, the M receptor blocker atropine (10^−6^ M, Sigma-Aldrich, St. Louis, MO), the purinergic antagonist, suramine hexasodium salt (10^−6^ M, Tocris Bioscience, UK) and the sodium-calcium exchanger (NCX) blocker, DCB (10^−5^ M, Sigma-Aldrich, St. Louis, MO) were used to explore the possible mechanism.

### Urodynamic evaluation in rats

Filling cystometry was performed according to previous reports^41^,^42^. After anesthetization via intraperitoneal injection of urethane (1 mg/kg), the bladders of the rats were exposed through a small median incision of lower abdomen. The dome of the bladder was punctured with a PE-50 catheter, and it was connected an infusion pump (AVI 270, 3 M, Minnesota, USA) and a multi-channel signal acquisition system (RM6480C, Chengyi, Chengdu, China) via a three-way pipe for both infusion and pressure recording. Room temperature saline or LCA (10^−6^ M) was infused at a rate of 0.2 ml/min. The continuous curves of intravesical pressure were recorded. The micturition interval and maximum bladder pressure were analyzed.

### Isolation of bladder SMCs

The bladder was put into 5 ml digestion solution and cut into approximately 1-mm^3^ pieces. The digestion solution contained 2.0 mg/mL type II collagenase, 2.0 mg/mL BSA, and 2.0 mg/mL trypsin inhibitor (all from Sigma-Aldrich, St. Louis, MO). The pieces were digested for approximately 35 min at 37 °C, and then 5 ml 10% fetal bovine serum was added to terminate the digestion. After centrifugation (1200 rpm/min for 5 min), the supernatant was discarded. Dulbecco’s modified Eagle’s media (DMEM) with 10% serum and 1% penicillin-streptomycin was added to the precipitate. The sample was beaten approximately 300 times with a dropper. The cells were filtered away from the tissue pieces with a 200-mesh (0.22 μm) cell strainer and seeded onto a glass coverslip at the bottom of a recording chamber. The SMCs were cultured in the incubator (37 °C, 95% O_2_ and 5% CO_2_).

### Detection of [Ca^2+^]_i_ using confocal microscopy

The cells were washed with PBS solution and dyed with Fluo-4/AM (10 μM, a cell-permeable fluorescent Ca^2+^ indicator) for 30 min in an incubator. Then, the Fluo-4/AM loaded cells were washed three times and allowed to stabilize for 10 min in hank’s balanced salt solution. Real-time images of [Ca^2+^]_i_ were collected at an emission wavelength of 488 nm using a Leica camera (Leica, Solms, Germany). The final data are presented as the relative fluorescent intensity (RFI): RFI = F1/F0, where F1 is the mean relative fluorescence intensity after drug administration and F0 is the mean baseline fluorescence intensity before drug administration. The experiments were carried out at room temperature.

### Recording the change in I_NCX_ induced by LCA using whole-cell patch clamp

The I_NCX_ was recorded in a whole-cell patch clamp. Patch pipettes were pulled from glass capillaries (1.5 mm outer diameter, 0.9 mm inner diameter; Narishige Scientific Instrument Laboratory, Tokyo, Japan) using a P-97 puller (Novato, CA, USA). The patch electrodes had a resistance of 3.0 MΩ–5.0 MΩ when filled with the internal solution (51 mM NaCl, 100 mM CsOH, 5 mM KCl, 2 mM MgCl, 20 mM tetraethylammonium Cl, 10 mM 4-(2-hydroxyethyl)-1-piperazineethanesulfonic acid (HEPES), 8 mM D-glucose, 1 mM adenosine 5′-triphosphate disodium salt hydrate (Na_2_ATP), 5 mM ethylene glycol tetraacetic acid (EGTA), 4.94 mM CaCl_2_; pH 7.2). The extracelluar solution contained 137 mM NaCl, 5 mM KCl, 1 mM MgCl_2_, 1.5 mM CaCl2, 10 mM HEPES and 10 mM D-glucose (pH 7.4). The I_NCX_ was induced by depolarization in 10 mV steps from −100 mV to +60 mV at a frequency of 1 Hz. The holding potential was set at −40 mV. Currents were recorded when SMCs were perfused in sequence with the extracellular solution, and solutions containing LCA (10^−6^ M) and Ni^2+^ (5 × 10^−6^ M) for 5 minutes, respectively. I_NCX_ was measured as the Ni^2+^-sensitive current that could be selectively inhibited by 5 × 10^−6^ M Ni^2+^. The data were recorded using EPC10 amplifiers (HEKA Electronics, Lambrecht, Germany). All of the experiments were conducted at 24 ± 2 °C.

### Statistical Analyses

The data are presented as the mean ± SD. All data were analyzed using the Statistical Package for Social Sciences, version 16.0, for Windows (SPSS, Chicago, IL). Statistical comparisons were performed using Student’s t-tests. All statistical tests were two-tailed. The level of statistical significance was set at P < 0.05.

## Additional Information

**How to cite this article**: Zhu, J. *et al.* Hydrophobic bile acids relax rat detrusor contraction via inhibiting the opening of the Na^+^/Ca^2^^+^ exchanger. *Sci. Rep.* **6**, 21358; doi: 10.1038/srep21358 (2016).
